# *SF3B1*^K700E^-driven transcriptional alterations in hematopoietic progenitors underlie blood cancer pathophysiology

**DOI:** 10.1016/j.gendis.2025.101775

**Published:** 2025-07-15

**Authors:** Mengzhu Xue, Jianhong An, Erqiang Hu, Wenjun Deng, Shanye Yin

**Affiliations:** a Innovation Center for AI and Drug Discovery, School of Pharmacy, East China Normal University, Shanghai 200062, China; b Department of Pathology, Albert Einstein College of Medicine, Bronx, NY 10461, USA; c Einstein Pathology Single-Cell & Bioinformatics Laboratory, Bronx, NY 10461, USA; d Montefiore Einstein Comprehensive Cancer Center, Albert Einstein College of Medicine, Bronx, NY 10461, USA; e Department of Neurology, Massachusetts General Hospital, Harvard Medical School, Boston, MA 02114, USA

Mutations in splicing factor 3b subunit 1 (*SF3B1*), particularly the K700E hotspot mutation, have been implicated in the pathogenesis of several hematological malignancies, including myelodysplastic syndromes (MDS), chronic lymphocytic leukemia, and acute myeloid leukemia. Despite the availability of various murine models for studying *SF3B1* mutations, there remains a notable discrepancy between the disease manifestations in these models and the human condition. Murine models often fail to fully recapitulate the spectrum of human blood cancers, particularly in terms of the transcriptional dysregulation observed in patients. This gap underscores the necessity for alternative models that can more accurately mirror the human disease phenotype to elucidate the underlying mechanisms of *SF3B1* mutations in oncogenesis.^1^ It is not understood what transcriptional dysregulation events are specifically induced by *SF3B1* mutation and are pivotal in the early stage of blood cancers.

Given the general role of *SF3B1* in splicing, a key unresolved question is why its mutation predominantly leads to blood cancers. Addressing this has been challenging, as previous studies were conducted in cancer or transformed cell lines. To delineate mutation-specific effects from those driven by the cancerous background, we introduced the K700E mutation into one allele of human embryonic stem cells (ESCs) using CRISPR engineering. Notably, this mutation induced the expression of hematopoietic genes in ESCs, which typically do not express these genes. These findings suggest that *SF3B1* mutations drive hematological malignancies by uniquely regulating hematopoietic gene expressions.

First, we utilized CRISPR technology to introduce the *SF3B1*^K700E^ mutation into human ESCs. Sanger sequencing confirmed the presence of this mutation in a heterozygous state, consistent with its occurrence in hematological malignancies (Fig. S1A). Both wild-type (WT) and *SF3B1*^K700E^ mutant (MT) ESC lines displayed normal karyotypes and similar colony morphologies (Fig. S1B and S1C). We then assessed the pluripotency of both cell lines through a teratoma formation assay, which confirmed their ability to differentiate into ectoderm, mesoderm, and endoderm tissues *in vivo* (Fig. S1D). Additionally, immunofluorescent staining verified the expressions of key stem cell markers, including OCT4, SSEA4, and TRA160, at similar levels in both lines (Fig. S1E). We induced ESC differentiation into hematopoietic progenitor cells (HPCs) to investigate the hematopoietic impact of the *SF3B1*^K700E^ mutation.

We performed RNA-sequencing analysis of WT and MT ESC lines. In MT cells, we detected the K700E mutation in SF3B1 mRNA, and the ratio between the MT transcripts and WT transcripts was 1:1 (Fig. 1A). We then analyzed significantly altered splicing events in MT cells versus WT cells. Together, we identified 1545 significant mis-splicing events (corrected *p*-value < 0.05) in MT cells compared with WT cells (Table S1). Notably, the majority of the aberrant splicing events involved reduced intron retention, followed by alternative 3′ splice site (3'ss) usage (Fig. 1B), indicating that *SF3B1*^K700E^ promotes usage of exons which were otherwise skipped in WT cells. One possibility is that these exons contain weak 3'ss unfavorable to WT *SF3B1* but preferentially used by *SF3B1*^K700E^. Considering that SF3B1 mutations are strongly associated with 3'ss mis-splicing, we investigated the occurrences of reported *SF3B1*^K700E^ alternative 3'ss events. Cryptic 3'ss involved well-known genes related to erythroid differentiation association (*FOXRED1*, *TMEM14C*, *PPOX*, and *STAU1*), RNA processing (*THOC1*), and canonical splicing target (*DVL2*) affected by *SF3B1*^K700E^ were identified (Fig. 1C). We validated alternative 3'ss splicing of *DVL2* gene, which plays critical role in Notch signaling pathway regulation (Fig. 1D). Notably, mis-spliced genes were highly enriched for tissue-specific genes expressed in various hematopoietic cell types (Fig. 1E). These results suggested that *SF3B1*^K700E^ induced mis-splicing events from key regulators of hematopoiesis, redox homeostasis, heme synthesis, mRNA stability, and blood malignancies, potentially driving transcriptional reprogramming in ESCs. This preferential effect on hematopoietic genes may explain why *SF3B1* mutations are strongly associated with hematological cancers.

**Figure 1:**
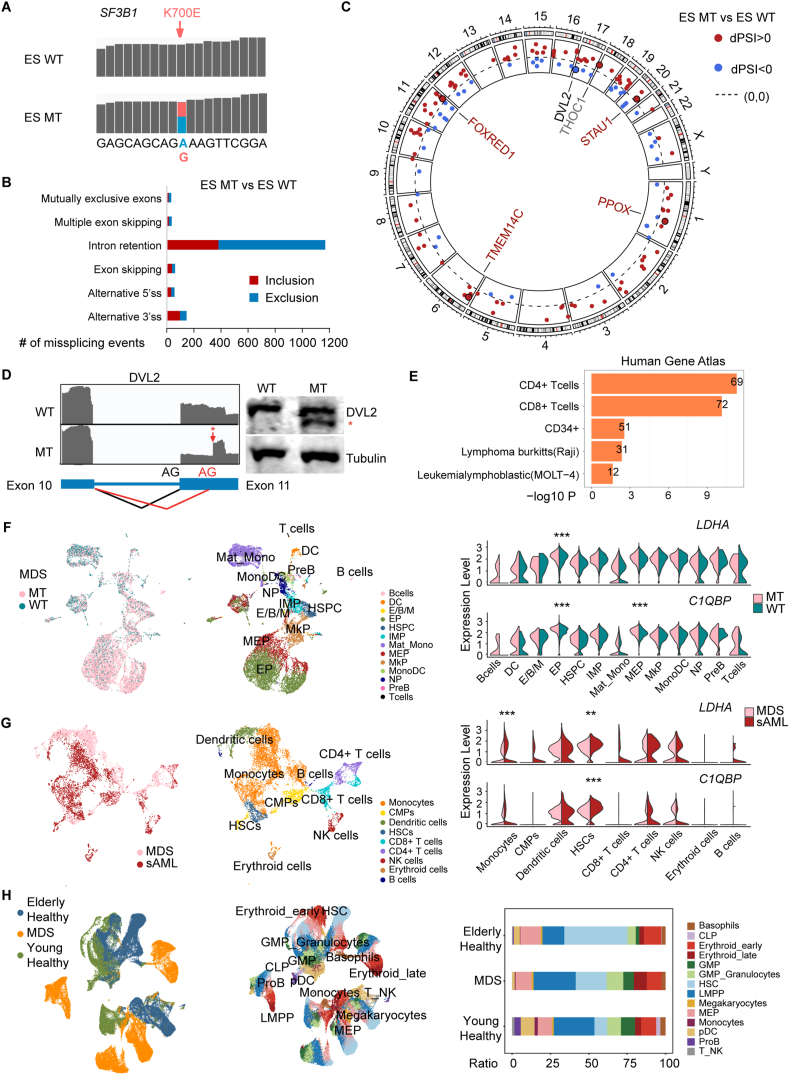
*SF3B1*^K700E^ mutation-induced transcriptional dysregulation events are associated with myelodysplastic syndromes (MDS) and acute myeloid leukemia (AML). **(A)** The ratio of mutant (MT) transcripts to wild-type (WT) transcripts in MT embryonic stem cells (ESCs) was determined by RNA sequencing. **(B)** Different categories of mis-splicing events detected in MT versus WT ESCs by the SplAdder algorithm. The events were retained with adjusted *p*-value < 0.05. Events with ΔPSI > 10% were defined as inclusion, and events with ΔPSI < 10% were defined as exclusion in MT versus WT ESCs. **(C)** Alternative 3′ splice site (3'ss) events detected in MT versus WT ESCs, shown with the hg38 genome by the circlize package. Cryptic 3'ss involved genes related to erythroid differentiation association (*FOXRED1*, *TMEM14C*, *PPOX*, and *STAU1*) and RNA processing (*THOC1*) in the publicly available dataset from MDS patients (GSE204845), and *DVL2* of canonical splicing affected by SF3B1 mutation. **(D)** Western blotting for 3'ss splicing of DVL2 in MT and WT ESCs. **(E)** Mis-splicing events associated with genes can be enriched as markers of hematopoietic cell types. The functional enrichment analysis was performed via the Enrichr website (https://maayanlab.cloud/Enrichr/). **(F)** Single-cell RNA-sequencing analysis was used to explore expression differences of signatured differentially expressed genes (DEGs) related to erythroleukemia and spliceosome in SF3B1^K700E^ MT versus WT hematopoietic cells from MDS patients. Cell-type assignments were from the public dataset of GSE204845 with annotations from Figure 1C.^3^ UMAP plots and violin plots were generated by the Seurat package. **(G)** Single-cell RNA-sequencing analysis was used to uncover expression differences of signatured DEGs related to erythroleukemia and spliceosome from secondary acute myeloid leukemia (sAML) versus MDS of the same patient (GSE205490).^4^ UMAP plots and violin plots were generated by the Seurat package. **(H)** Single-cell RNA-sequencing analysis was used to indicate ratio variations of erythroid late cells and hematopoietic stem cells from young healthy donors, elderly healthy donors, and MDS patients. Cell-type assignments were from the public dataset of GSE180298 with annotations from Figure 1C.^5^ UMAP plots and bar plots were generated by the Seurat package and the ggplot2 package.

We further performed RNA-sequencing analysis to explore transcriptional dysregulation events in MT versus WT HPCs. The analysis confirmed the *SF3B1*^K700E^ mutation at a 1:1 ratio of MT to WT transcripts in MT HPCs (Fig. S2A). Our analysis identified several significant splicing alterations, predominantly intron retention, exon skipping, and alternative 3'ss in MT versus WT HPCs (Fig. S2B; Table S2). Comparative analysis with ESCs highlighted transmembrane protein 14C (*TMEM14C*), which showed 3'ss mis-splicing in both MT HPCs and MT ESCs (Fig. S2C). The usage of the *TMEM14C* 3'ss mis-splicing site was shown on the hg38 genome and further validated by reverse transcription PCR (Fig. S2D–S2F). *TMEM14C* is related to erythroid differentiation and ring sideroblast formation in MDS,^2^ which exhibits distinct splicing patterns in MDS, underscoring their potential roles in erythroid differentiation and disease pathology. These results reveal the *SF3B1*^K700E^ mutation induces mis-splicing events associated with MDS involvement and erythroid differentiation, and indicate the potential of the *TMEM14C* 3'ss mis-splicing as a diagnostic biomarker for MDS at the early stages.

In our exploration of the global gene expression impacts of the *SF3B1*^K700E^ mutation in HPCs, we observed significant changes: 1887 genes were up-regulated and 2033 were down-regulated by > 1.5-fold (false discovery rate < 0.05) (Fig. S2G). Notably, 111 of these differentially expressed genes were also identified as mis-spliced genes implicated in erythroleukemia (Fig. S2H). A deeper analysis of these 111 genes revealed that most were down-regulated in MT versus WT HPCs (Fig. S2I), including erythroleukemia genes like *LDHA* (a key enzyme in anaerobic glycolysis) and spliceosome-related genes like *C1QBP* (a mitochondrial protein). Given the link between *SF3B1*^K700E^ mutation-induced mis-splicing and MDS, we analyzed the single-cell RNA-sequencing data^3^ from WT and *SF3B1*^K700E^ MDS patients and observed notably higher expression levels of *LDHA* and *C1QBP* in MT erythroid progenitor cells and *C1QBP* in megakaryocytic-erythroid progenitor cells (Fig. 1F). Further analysis from an MDS patient with secondary acute myeloid leukemia^4^ showed elevated *LDHA* and *C1QBP* expression in hematopoietic stem cells and monocytes (Fig. 1G). Lastly, we analyzed the single-cell RNA-sequencing dataset from young healthy donors, elderly healthy donors, and MDS patients,^5^ and found that the ratios of erythroid late cells to hematopoietic stem cells varied greatly (Fig. 1H). Comparisons with healthy donors highlighted significant variations in cell ratios, suggesting that the *SF3B1*^K700E^ mutation leads to distinct gene expression patterns in MDS, contrasting with those in normal HPCs. These findings demonstrate that the *SF3B1*^K700E^ mutation triggers differential expression of key genes involved in erythroleukemia and the spliceosome. The regulatory effects vary between HPCs and MDS progression, with opposite directions of gene regulation observed in these contexts. The discrepancy may suggest distinct molecular mechanisms at play in different stages, contexts of the disease, microenvironmental influences, and disordered energy metabolism in the pathological state. Understanding these differences will lead to more targeted and effective therapeutic strategies, tailored to the specific pathological features of MDS at various stages.

In conclusion, our study establishes the utility of our ESC/HPC model as a robust platform for investigating the oncogenic transcriptional dysregulation caused by the *SF3B1*^K700E^ mutation. We demonstrate that this mutation alters the splicing patterns and expression levels of key genes involved in erythroleukemia and spliceosome dynamics, suggesting the potential of the *TMEM14C* 3'ss mis-splicing as an early-stage diagnostic biomarker for MDS. Furthermore, the observed regulatory patterns in HPCs differ markedly from those in cells from MDS patients, highlighting the importance of studying the *SF3B1*^K700E^ mutation in the early stages of blood cancer development. This not only advances our understanding but also underscores the potential for targeted early interventions.

## CRediT authorship contribution statement

**Mengzhu Xue:** Visualization, Conceptualization, Methodology, Writing – review & editing, Writing – original draft, Data curation. **Jianhong An:** Methodology, Data curation. **Erqiang Hu:** Methodology, Data curation. **Wenjun Deng:** Conceptualization, Writing – review & editing. **Shanye Yin:** Writing – original draft, Funding acquisition, Project administration, Data curation, Writing – review & editing, Conceptualization, Methodology.

## Funding

This work was in part supported by the 10.13039/100000002US National Institutes of Health *R*21 CA267527-01 (to S.Y.).

## Conflict of interests

W.D. and S.Y. hold equity in Yihui Bio, Inc. All other authors declare no potential conflict of interests.
