# Supplementary material for: SF3B1K700E-driven transcriptional alterations in hematopoietic progenitors underlie blood cancer pathophysiology
Source: Genes Dis. 2025 Jul 15;13(4):101775. doi: 10.1016/j.gendis.2025.101775 (PMC13011019; doi:10.1016/j.gendis.2025.101775)
Supplement: Multimedia component 1 [file mmc1.docx]

**Material and Methods**

*CRSPR engineering of SF3B1^K700E^ mutation into ES cells*

The H9 ES cell line (WA09) was obtained from WiCell Research Institute, Inc., Madison, WI. ES cells were cultured in in E8 medium ^1^ on Matrigel-coated tissue culture plates at 37 °C with 5% (vol/vol) CO_2_. CRISPR/CAS9-stimulated homology-mediated repair was used to generate isogenic ES cell lines containing an A>G point mutation resulting in SF3B1^K700E^ amino acid substitution. CRISPR guide sequence: 5’-TGGATGAGCAGCAGAAAGTT-3’; Ultramer sequence used to introduce mutation (point mutation uncapitalized (AAA to gAA); PAM region underscored): 5’-TGTAACTTAGGTAATGTTGGGGCATAGTTAAAACCTGTGTTTGGTTTTGTAGGTCTTGTGGATGAGCAGCAG**g**AAGTTCGGACCATCAGTGCTTTGGCCATTGCTGCCTTGGCTGAAGCAGCAACTCCTTATGGTATCGAATCTTTTGAT-3’. Single colonies were screened for their mutation status using a high-throughput screening strategy involving barcoded amplification of the targeted region of *SF3B1* and next-generation sequencing. The clone with *SF3B1* mutation was further verified by Sanger sequencing as well as by RNA-Seq. The teratoma formation assay was performed according to previous method. ^2^

*HPC cell differentiation*

To induce the differentiation of ES cells into HPCs, the STEMdiff™ Hematopoietic Kit (# 05310, STEMCELL TECHNOLOGIES) was used according to the manufacturer's Protocol. The HPCs were defined phenotypically as CD34⁺CD43⁺ cells, which represent a population enriched for early hematopoietic progenitors derived from pluripotent stem cells. Following differentiation, cells were harvested at Day 12–15, and CD34⁺CD43⁺ HPCs were isolated by fluorescence-activated cell sorting (FACS) for downstream experiments.

Western blotting on DVL2 isoforms

The western blotting experiment to assess DVL2 isoforms were performed according to previous methods. ^3^

*RT-PCR of TMEM14C isoforms*

We performed RT-PCR to validate the alternative 3’ splice site (3’ ss) usage in TMEM14C. RT-PCR primers: F-GACACCTCGCAGTCATTCCT, R-TGATCCCACCAGAAGCAACC.

*Bulk RNA-seq determination and data analyses*

Total RNA extracted and poly-A selected with bulk RNA-seq libraries were described as previous report. ^4^ Fastq data were aligned to the hg38 genome by STAR (v 2.7.8a), using the 2-pass mode with parameters recommended by the TCGA. ^5^ Alternative splicing analysis was performed using SplAdder ^6^ with parameters recommended by the TCGA.^5^ Then, events were filtered by adjusted p value < 0.05 and |ΔPSI| > 0.1. Circos plots were generated by the circlize package (v 0.4.15) in R 4.2. RNA-seq coverage plots were generated in the IGV tool (v 2.13.0). Expression levels were quantified by TPM values and filtered as described previously. ^3^ DEGs were analyzed by the edgeR package (v 3.42.4) in R 4.2. The Enrichr website (https://maayanlab.cloud/Enrichr/) were used for functional enrichment analysis.

*ScRNA-seq analyses*

Data matrix, barcode, and metadata files were downloaded from the GEO database, with accession numbers of GSE204845, GSE205490, and GSE180298. The Seurat package (v 5.0) in R 4.2 was used to convert h5 files into Seurat objects and do data merging, scaling, clustering, and plotting. Cell types were assigned according to the metadata file in GSE204845 and GSE180298, respectively. For GSE205490, cell types were assigned by the SingleR package (v 2.0.0) in R 4.2 with the reference of Novershtern Hematopoietic Data.

**References**

1 Chen, G. *et al.* Chemically defined conditions for human iPSC derivation and culture. *Nature methods* **8**, 424-429 (2011). <https://doi.org/10.1038/nmeth.1593>

2 Lensch, M. W., Schlaeger, T. M., Zon, L. I. & Daley, G. Q. Teratoma formation assays with human embryonic stem cells: a rationale for one type of human-animal chimera. *Cell Stem Cell* **1**, 253-258 (2007). <https://doi.org/10.1016/j.stem.2007.07.019>

3 Wang, L. *et al.* Transcriptomic Characterization of SF3B1 Mutation Reveals Its Pleiotropic Effects in Chronic Lymphocytic Leukemia. *Cancer Cell* **30**, 750-763 (2016). <https://doi.org/10.1016/j.ccell.2016.10.005>

4 Landau, D. A. *et al.* Evolution and impact of subclonal mutations in chronic lymphocytic leukemia. *Cell* **152**, 714-726 (2013). <https://doi.org/10.1016/j.cell.2013.01.019>

5 Kahles, A. *et al.* Comprehensive Analysis of Alternative Splicing Across Tumors from 8,705 Patients. *Cancer Cell* **34**, 211-224.e216 (2018). <https://doi.org/10.1016/j.ccell.2018.07.001>

6 Kahles, A., Ong, C. S., Zhong, Y. & Rätsch, G. SplAdder: identification, quantification and testing of alternative splicing events from RNA-Seq data. *Bioinformatics* **32**, 1840-1847 (2016). <https://doi.org/10.1093/bioinformatics/btw076>


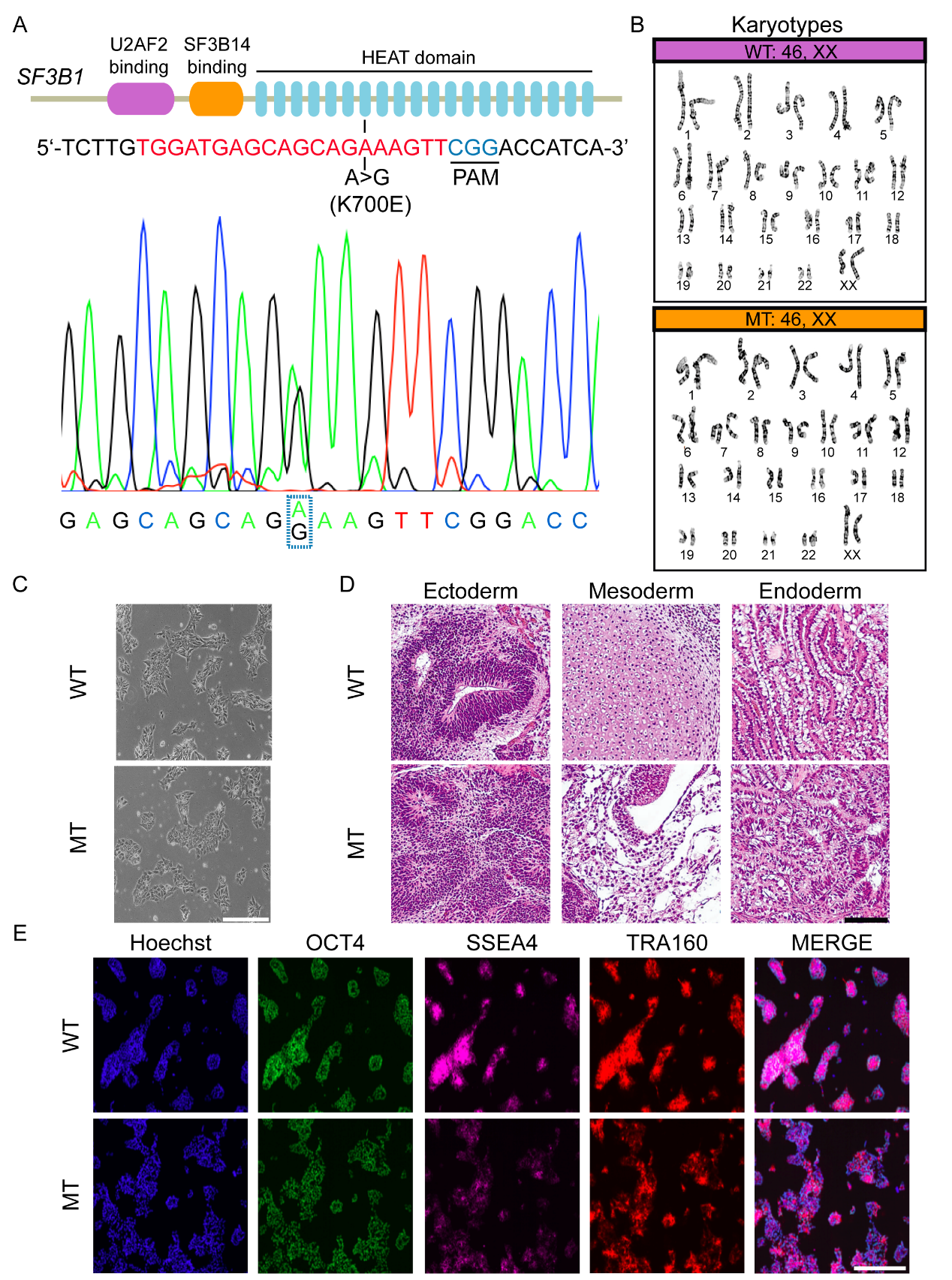


**Figure S1** CRSPR engineering of SF3B1K700E mutation into human embryonic stem cells. (**A**) Schematic of *SF3B1* showing the domain structure and the position of the K700E mutation. Target sequence is labeled red followed by a protospacer adjacent motif (PAM) labeled blue. Sanger sequencing result shows that the K700E mutation was successfully engineered into one allele of the ES cells. (**B**) Karyotyping results showed that WT and MT ES cells had normal karyotypes. (**C**) Cellular morphology results showed that WT and MT ES cells had similar cellular morphologies. Scale bar, 50 μm. (**D**) We then used a teratoma formation assay to evaluate the pluripotency of WT and MT ES cells. Hematoxylin and Eosin (H&E) staining results of ectoderm, mesoderm, and endoderm tissues. Scale bar, 50 μm. (**E**) Immunofluorescent (IF) staining results of stem cell markers from WT and MT ES cells, using antibodies of OCT4, SSEA4, and TRA160. Scale bar, 50 μm.


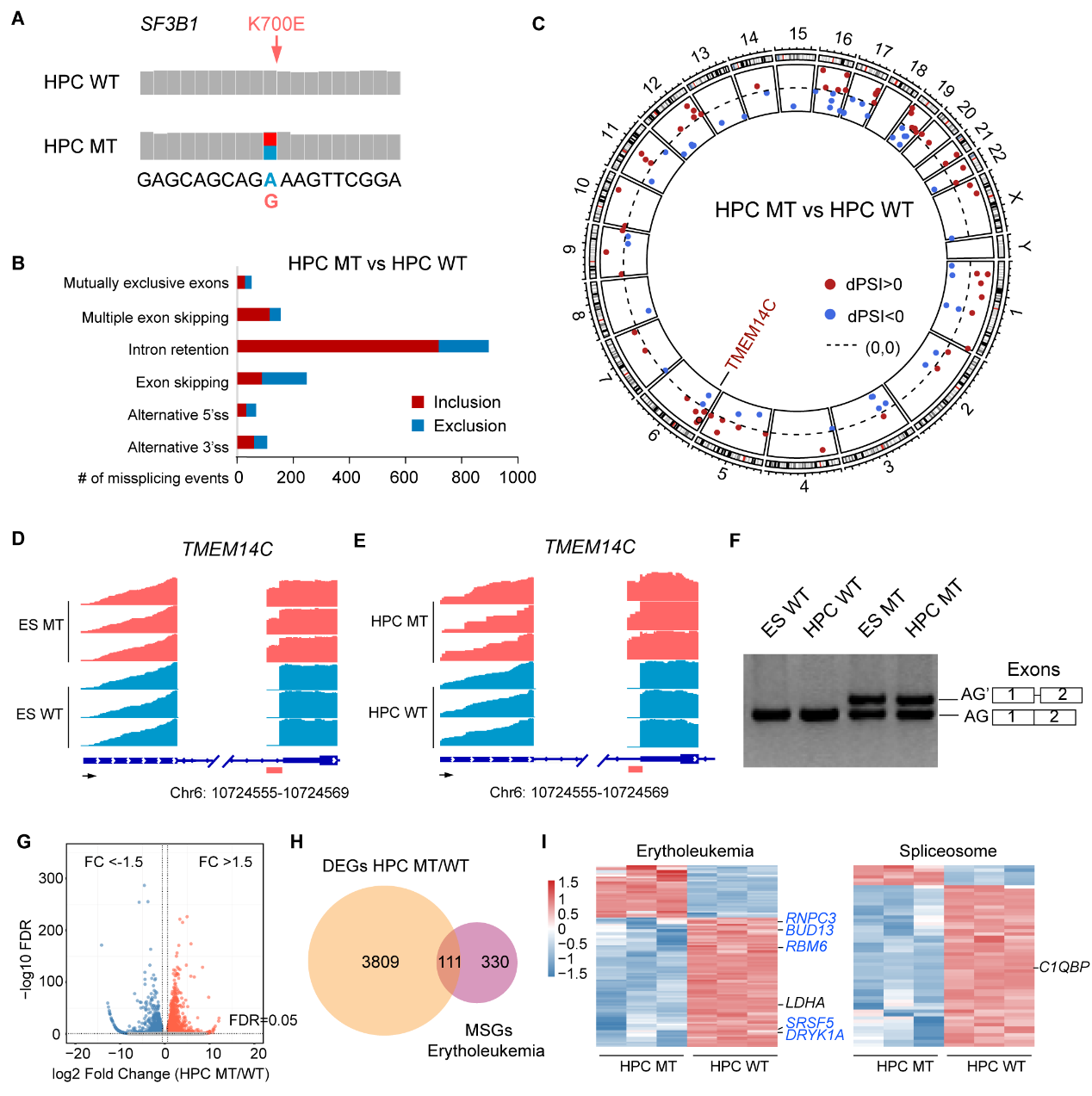


**Figure S2** SF3B1K700E mutation altered splicing patterns and expression levels of genes involved in erythroleukemia and the spliceosome. (**A**) Ratio of MT transcripts and WT transcripts in MT HPCs, determined by the RNA-seq. (**B**) Different categories of mis-splicing events detected in MT versus WT HPCs are shown, in which events were retained with adjusted p value < 0.05. Events with ΔPSI > 10% were defined as inclusion and events with ΔPSI < -10% were defined as exclusion in MT compared with WT HPCs. (**C**) Cryptic 3’ splice sites of *TMEM14C* in MT versus WT HPCs, shown with the hg38 genome. (**D** and **E**) The coverage of the *TMEM14C* 3’ss splice site in MT vs WT ES cells (**D**) and HPCs (**E**), shown by the IGV software on the hg38 genome. (**F**) The RT-PCR results of the *TMEM14C* 3’ss splice site in ES WT, HPC WT, ES MT, and HPC MT cells. (**G**) DEGs in MT versus WT HPCs, which were retained with FDR < 0.05 and |fold change| > 1.5. (**H**) Venny’s plot to show the intersection between DEGs and MSGs related to erythroleukemia. (**I**) Gene expression profiles of intersected DEGs related to erythroleukemia and spliceosome signatures in MT and WT HPCs.

**Table S1** Mis-splicing events detected in mutant (MT) versus wild-type (WT) embryonic stem cells (ESCs).

**Table S2** Mis-splicing events detected in mutant (MT) versus wild-type (WT) hematopoietic progenitor cells (HPCs).
